# Inducing preference reversals in aesthetic choices for paintings: Introducing the contrast paradigm

**DOI:** 10.1371/journal.pone.0196246

**Published:** 2018-04-19

**Authors:** Zorry Belchev, Glen E. Bodner, Jonathan M. Fawcett

**Affiliations:** 1 Department of Psychology, University of Calgary, Calgary, Alberta, Canada; 2 Department of Psychology, Memorial University of Newfoundland, St. John’s, Newfoundland, Canada; Coventry University, UNITED KINGDOM

## Abstract

Understanding what leads people to reverse their choices is important in many domains. We introduce a contrast paradigm for studying reversals in choices—here between pairs of abstract paintings—implemented in both within-subject (Experiment 1; N = 320) and between-subject (Experiment 2; N = 384) designs. On each trial, participants chose between a pair of paintings. A critical pair of average-beauty paintings was presented before and after either a reversal or control block. In the reversal block, we made efforts to bias preference away from the chosen average-beauty painting (by pairing it with more-beautiful paintings) and toward the non-chosen average-beauty painting (by pairing it with less-beautiful paintings). Meta-analysis revealed more reversals after reversal blocks than after control blocks, though only when the biasing manipulations succeeded. A second meta-analysis revealed that reversals were generally more likely for participants who later misidentified their initial choice, demonstrating that memory for initial choices influences later choices. Thus, the contrast paradigm has utility both for inducing choice reversals and identifying their causes.

## Introduction

Making choices is a ubiquitous part of daily life. Evaluations are often made impetuously [1], yet they can have enduring effects on our preferences [2]. An important research area is thus to understand how experiences shape our preferences, that is, our liking of one stimulus over another [3–6]. To this end, here we introduce a contrast paradigm for inducing reversals in choices between pairs of stimuli. Our paradigm also enables the exploration of predictors of reversals, such as the accuracy of one’s memory for prior choices.

Preferences have been studied for stimuli ranging from faces [7] to music genres [8] to political views [9]. Here we investigated aesthetic judgments (i.e., those concerned with one’s appreciation of beauty), an area rich in history in psychology [10]. Specifically, we measured reversals in aesthetic choices among pairs of abstract paintings that were matched for beauty and initial preference via piloting. Although art appreciation is colloquially believed to be highly subjective, there is good agreement regarding what makes a painting beautiful (e.g., greater complexity, semantic meaning) [11], and people’s preferences are relatively consistent [12]. Of course, people may be more likely to reverse their choice when a preference has not yet been established, and/or when they initially deem the two choices to be equivalent. Before introducing our contrast paradigm, we briefly review existing methods of producing preference reversals.

Four methods have commonly been used to induce preference reversals, and subsequently to enable the factors that moderate reversals to be studied. First, task-induced preference reversals can be induced by changing the nature of the task across choices. This method is often embedded in gambling tasks in which participants are first asked to choose between options, and are later asked to bid on each option [13, 14]. Second, frame-induced preference reversals can be induced by framing two equivalent options in opposite ways for different sets of participants. For example, as pioneered by Tversky and Kahneman [15], framing disease treatment-program choices in terms of “lives lost” leads to a preference for risk-seeking programs, whereas framing in terms of “lives saved” leads to a preference for risk-averse programs. Third, context-induced preference reversals can be induced by manipulating the availability of other options and their relative inferiority/superiority [16]. This method often introduces a third option that alters one’s evaluation of the other two options, usually termed a target and a competitor [17–21]. These three methods are usually used to study reversals in value-based choices and ratings associated with extrinsic rewards or consequences (i.e., gambling), or between consumer products. More recently, context-dependent methods have also been used to influence riskless, perception-based judgments not associated with explicit rewards or losses (e.g., choosing which shape has the largest area), showing that context influences many types of decisions [22–24]. A fourth method for inducing reversals for such judgments relies on a gaze bias manipulation in which after making an initial choice, the stimulus not chosen is presented for longer durations than the chosen stimulus during cycles of an exposure phase. On a second choice trial, this gaze bias can induce a choice reversal [25–26].

Our study departed from these methods of inducing preference reversals. We did not manipulate the task, the framing of the choices, the choices offered, or how those choices were presented. Instead, we simply manipulated the choices participants made on other trials involving the critical paintings. Specifically, we attempted to induce a choice reversal for the same pair of paintings merely by inserting a set of 6 choice trials between the two presentations that capitalized on the existence of contrast effects on aesthetic judgments.

When context influences a judgment, either contrast or assimilation may result [27]. When considering aesthetic judgments, a contrast effect occurs when a target stimulus (e.g., a painting) is liked more when presented in the context of a less-pleasant stimulus (e.g., a less-beautiful painting), and/or is liked less when presented in the context of a more-pleasant stimulus (e.g., a more-beautiful painting). An assimilation effect refers to the converse outcome: A target painting is liked more when presented with a more-beautiful painting, and/or is liked less when presented with a less-beautiful painting.

Perceptual contrast effects include the Ebbinghaus illusion in which circles of the same size are perceived as smaller if they are surrounded by big circles, or bigger if they are surrounded by smaller circles [28]. In the realm of aesthetics, a musical melody was liked less when presented after a more-pleasant melody than when it was presented first (i.e., a negative contrast effect) [29]. When the more-pleasant musical melody was presented second, it was liked more than if it had been presented first (i.e., a positive contrast effect). Some researchers have found that evaluations of facial beauty can evoke assimilation effects [30]. To date, however, context has predominantly yielded contrast rather than assimilation effects on aesthetic judgments. For example, contrast effects occur for photographs [31], representational paintings [32], and abstract paintings [33–35].

We developed a contrast paradigm designed to induce reversals in people’s aesthetic choices between pairs of average-beauty abstract paintings. Through a pilot study, we selected average, low, and high beauty paintings for use in the paradigm. At the outset of the main experiments, participants chose which of two average abstract paintings from a critical pair was deemed more beautiful. In a reversal block, we attempted to shift their preference from their initially chosen painting toward the other painting. To this end, on some reversal block trials, participants chose between their initial choice and a more-beautiful painting—thus we used contrast to decrease the perceived beauty of their initial choice painting. On other trials, participants chose between the non-chosen critical painting and a less-beautiful painting—thus here we used contrast to increase the perceived beauty of their non-chosen painting. The same pair of critical paintings was then presented again. Our measure was whether a preference reversal occurred across choices. This reversal rate was compared to a control block in which the same trials described above involved a different pair of average-beauty paintings. To test for generality, this contrast paradigm was implemented in a within-subjects design in Experiment 1A/1B (i.e., each participant received 1 reversal block and 1 control block) and in a between-subjects design in Experiment 2 (i.e., each participant received 2 reversal blocks or 2 control blocks). The between-subject design increased the number of choice reversal opportunities for each condition, and eliminated the potential carry-over effects of the preceding block type.

We collected data in a small number of experiments using large samples. This was necessary because each experiment had only 1 or 2 critical pairs for a given block type, giving us only 1 or 2 opportunities to observe a choice reversal for each participant. In turn, the number of blocks we could present was constrained by the number of consistently rated paintings we were able to obtain. To provide the most accurate picture of the pattern of results from these experiments we therefore used a meta-analytic approach, which allowed us to explore whether a reversal effect occurred, and also whether the use of a within- vs. between-subject design moderated this effect. Another potential moderator of choice reversals we examined through meta-analysis was whether reversals were more likely when participants chose the higher-beauty paintings within the blocks. This possibility seemed likely given that if our attempts to bias their interim choices were not effective, then we should not expect to obtain a preference reversal. Regardless of whether the contrast paradigm succeeded, some reversals will occur for both reversal and control blocks, particularly given the matching of initial preference for the critical painting pairs. Importantly, the contrast paradigm is useful for examining predictors of reversals other than contrast. Experiments 1B and 2 examined a third potential moderator of reversals highlighted in recent studies: People’s memory for their initial choices. A single choice or rating of an item among other alternatives has been found to affect later judgments/choices involving that item, by strengthening the initial assessment of that item and weakening the assessment of the alternatives [36–37]. Such post-decision changes have been argued to result from memory consolidation and increased differentiation between chosen and rejected items [37–38]. It has also been linked with a desire to reduce cognitive dissonance [39], wherein providing negative information about chosen products following initial assessments can induce greater change in favor of the chosen item than providing positive information [36, 40].

More recently, choice-induced preference changes have been reported using a paradigm that incorporates two consecutive rating opportunities for the same items, to control for artifacts caused by regression toward the mean [41–43]. Using this paradigm, Salti et al. [43] first had participants rate their desire to visit each of a set of vacation destinations (rating opportunity 1). A forced-choice block presented pairs of a subset of the previously rated destinations (choice block 1). Another rating of all of the destinations then occurred (rating opportunity 2), followed by a final choice block for the remaining subset of destinations (choice block 2). The second ratings of items from choice block 1 represent the uncorrected rating-choice-rating condition, whereas those from choice block 2 represent the rating-rating-choice condition. Ratings for previously chosen vacations increased across rating opportunity 1 and 2 in both conditions. Genuine preference changes have also been reported when the rating scale changed across rating opportunities from liking to willingness-to-pay [44].

Memory for previous judgments has been shown to moderate these preference changes. For example, participants who forget their initial choice are significantly less likely to exhibit the expected spread of alternatives across ratings [41, 43]. The authors of these studies propose that forgetting one’s initial choices eliminates the possibility of experiencing cognitive dissonance later on, such that participants no longer feel pressured to strengthen their initial judgments by increasing their second ratings. In line with this cognitive dissonance explanation, participants who remember their initial choice in our contrast paradigm may also feel more pressure to later make a consistent choice. To evaluate this possibility, in Experiment 1B and 2 we measured people’s subsequent memory for their initial choice. We then used meta-analysis to determine whether accuracy of memory for initial choice moderated preference reversals. Based on prior findings [41, 43], we expected that this would indeed be the case.

## Experiment 1: Within-subject design

### Method

#### Ethics statement

This research was approved by the Conjoint Faculties Research Ethics Board at the University of Calgary. Participants received course credit in a psychology course in exchange for participating. They gave informed consent for the online study by reading an on-screen consent form and clicking an “I agree to participate” button. An on-screen debriefing was provided at the end of the study.

#### Participants

Undergraduates from a research participation pool volunteered to take part in either Experiment 1A (*N* = 96, female = 62, mean age = 21, age range = 18–37) or Experiment 1B (*N* = 224, female = 166, mean age = 21, age range = 17–52). Only 4 participants identified as art experts post-experiment, so art expertise was not considered further.

#### Stimulus selection

The stimuli were abstract paintings selected through four initial pilot studies, each of which used unique additional sets of participants from the same participation pool. At the outset, 240 images of abstract paintings were chosen from several online image databases (e.g., Artstor, Oxford Art Online) and Google searches (e.g., “ugly paintings”) in an effort to span a wide range of beauty. To obtain enough high-beauty paintings it was necessary to include a few paintings by somewhat well-known artists (e.g., O’Keefe), but all or nearly all of paintings were unfamiliar to the participants, particularly given that the vast majority of them self-identified as art novices. The selected paintings typically did not depict obvious semantic or representational content. Each image was resized to 500 pixels on the longer dimension. An initial 21 participants rated the beauty of all 240 paintings on a 9-point scale (1 = ugly, 5 = neither ugly nor beautiful, 9 = beautiful). Based on their ratings, 36 paintings were selected that were rated as close to 1 (low), 5 (average), and 9 (high) as possible, each of which had a standard error below 0.5. Another 23 participants rated the beauty of these 36 paintings twice (in successive blocks, freshly randomized for each participant and block). The mean of their two ratings was used to select the final set of 24 paintings using the same criteria. Another 22 participants rated the final set twice. The mean for each set, again based on the mean of the two ratings was 3.18 (*SD* = .31) for low-beauty paintings, 5.06 (*SD* = .26) for average-beauty paintings, and 6.78 (*SD* = .20) for high-beauty paintings. The correlation between the two ratings for a given participant (averaged across paintings) was .85, and the correlation between the two ratings for a given painting (averaged across participants) was .80. Thus, the ratings were quite stable by participants and by paintings. Finally, another 80 participants were shown 3 or 4 average/average pairs, 8 average/low pairs, and 8 average/high pairs. They were asked to choose the more-beautiful painting from each pair. The pairs were then presented a second time in a freshly randomized order. Based on their choices, 4 average/average pairs were selected to elicit as close to a 50/50 split in choices as possible (*M* = .48), 32 average/low pairs were selected to maximize the proportion of average-beauty choices (*M* = .89), and 32 average/high pairs were selected to maximize the proportion of high-beauty choices (*M* = .91). The painting images, due to copyright reasons, are available from the first author.

#### Design

**Fig 1:**
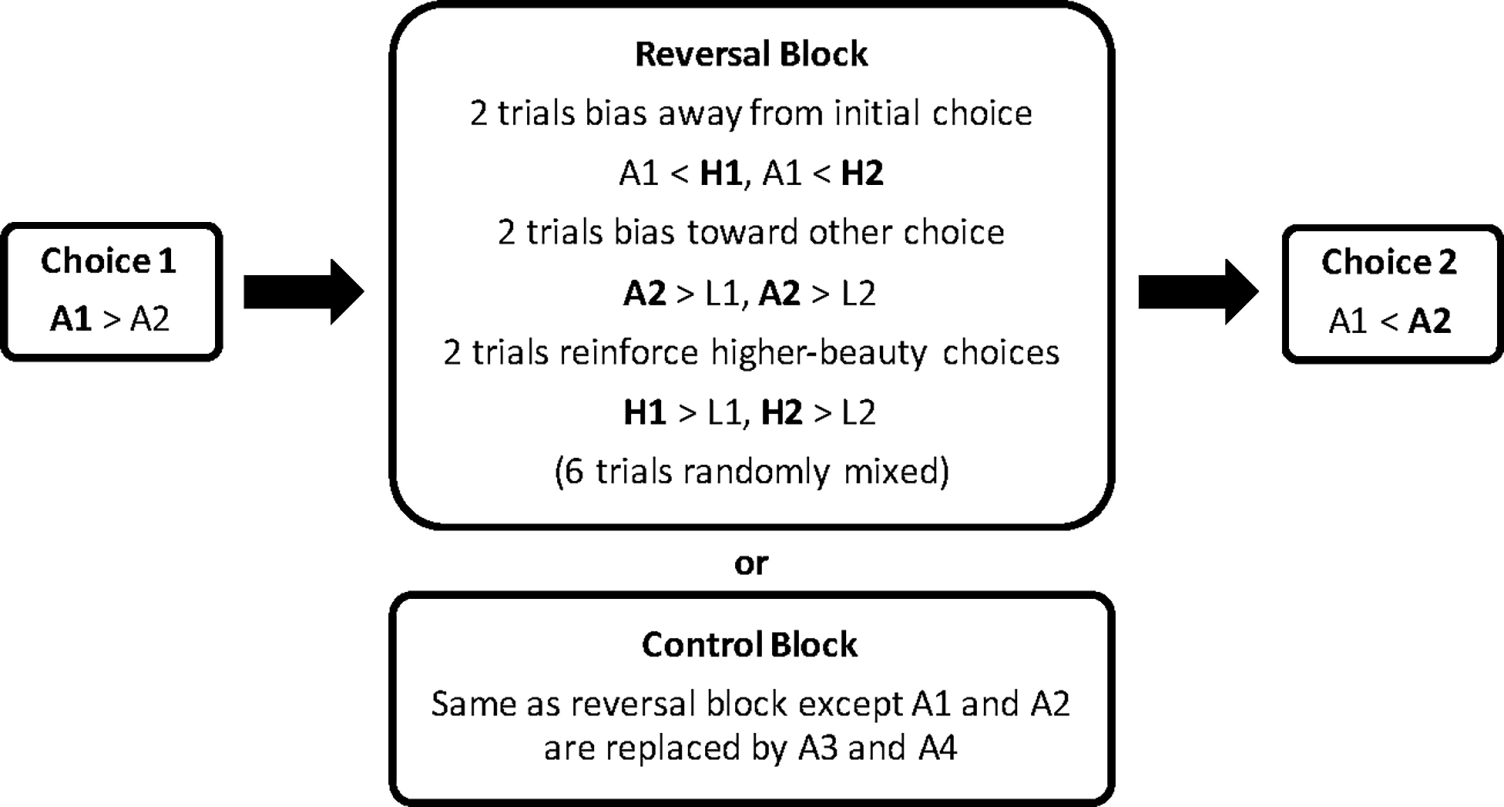
The contrast paradigm. Reversal and control blocks were bookended by a choice between the same two average-beauty paintings, denoted A1 and A2. The example illustrates a preference reversal because A1 was chosen for Choice 1 but A2 was chosen for Choice 2. L and H refer to low- and high-beauty paintings, respectively. Numbers denote different paintings.

In Experiment 1, each participant received one reversal block and one control block, the order of which was counterbalanced across participants. There was no overlap in the paintings shown in the two blocks. To measure reversals, the same critical pair of average paintings was presented before and after each block (Fig 1). The left/right order of the critical painting pairs always changed across choices. A filler block (not analyzed), consisting of two high/low painting pairs not part of the final painting sets, was presented before choice 1 in each block both to provide task practice and to help mask the structure of the main trials.

The reversal and control blocks each consisted of 6 randomly ordered trials, bookended by the critical average-beauty pair (Fig 1). Choice 1 dictated the structure of the reversal block. The reversal block included 2 trials in which the non-chosen average painting was paired with a low-beauty painting. These trials were designed to bias participants toward the painting they did not pick for choice 1. The reversal block also included 2 trials in which the chosen average painting was paired with a high-beauty painting. These trials were designed to bias participants away from the painting they picked for choice 1. The inclusion of both types of contrast trials (biasing toward non-chosen paintings and away from chosen paintings) ensured matched exposure to both the chosen and non-chosen painting from choice 1 and thus eliminated mere exposure as a potential confounding factor [45]. There were also 2 high/low-beauty pair trials. These trials reinforced choosing the higher-beauty paintings while controlling the number of exposures to each low, average, and high-beauty painting (i.e., 2 exposures each). Each painting appeared once on the left side and once on the right side.

The control block was identical to the reversal block, except a different pair of average-beauty paintings, not presented elsewhere in the experiment, was used in the control block sequence (Fig 1). The control block was not expected to impact choice 2 because neither critical painting appeared in the control block. The critical test of the reversal effect was whether the reversal rate was higher in the reversal blocks than in the control blocks. In total, each participant received 20 pairs of paintings.

Assignment of average/average pairs to block type (reversal vs. control) and role (reversal pair vs. control pair) was counterbalanced. Each participant received the 2 low-beauty and 2 high-beauty paintings within a given block, but the assignment of average painting pairs and block type were counterbalanced. The 8 counterbalances were randomly assigned to participants. S1 Table provides the counterbalancing details for each experiment.

#### Procedure

Participants took part in the online study through the Department’s online research participation site. The instructions told participants to allot 30 minutes to complete the experiment in one session, but no time limit was placed on their responses or session. They were asked to maximize their browser window to enable them to view the full paintings without scrolling, and they were asked to limit distractions by exiting other applications and putting away their phones. They were then given the following instructions:

On each trial in this study you will be shown two paintings side by side. Your task is to choose which painting you think is more beautiful. Make your choice based on your automatic and spontaneous feelings for the two paintings at that moment. Recommended ways to choose between the two paintings include imagining which painting you would like to see again, or which painting you would most prefer to hang on your wall. You will be shown some pairs of paintings twice because we wish to determine whether what people deem beautiful in art is stable or variable over time. You should not feel any pressure to choose the same painting each time, nor should you feel any pressure to choose a different painting each time. Instead, each time you see a pair of paintings just choose based on your automatic and spontaneous feelings for each painting at that moment.

These instructions were designed to avoid biasing participants either toward or away from reversing their choices.

Experiments 1A and 1B were identical with two exceptions. First, Experiment 1B also considered whether reversals were associated with memory for choice 1 by measuring initial-choice identification accuracy. To this end, after the second block, participants were shown the critical average-beauty pair from each block again, starting with the critical pair from their more recent block. They were asked to “indicate which painting you chose when you were shown this exact pair of paintings in this order earlier in the study.” The paintings appeared in the same left/right order as on the choice 1 trial, to help cue participants’ memory. Second, we were also interested in determining whether reversals might be more likely when participants had a weaker preference for choice 1. Therefore, in Experiment 1B, after each choice in each block, participants indicated on a 9-point scale their degree of preference for the chosen versus non-chosen painting (1 = slightly more, 5 = somewhat more, 9 = very much more) [46]. These ratings proved uninformative and are not discussed. The mean completion time was 6.24 min in Experiment 1A and 10.37 min in Experiment 1B; no participants exceeded 30 minutes.

## Experiment 2: Between-subject design

### Method

Another unique set of participants were selected from the same participation pool (*N* = 384, female = 294, mean age = 20, age range = 17–58); a larger sample was drawn due to the between-subject design. Participants were assigned to either the reversal or control group and to a given counterbalance in equal numbers (see S1 Table). Experiment 2 was identical to Experiment 1B except participants received either 2 reversal blocks or 2 control blocks. The mean completion time was 9.28 min; 8 participants were replaced for exceeding 30 min.

### Results

The data and meta-analyses can be found in the following OSF repository: https://osf.io/j5a76/. Three meta-analyses were conducted on the raw data from all participants to maximize power while allowing us to examine the potential moderation of our effect. Models were fit using the rstanarm package [47] in *R 3*.*3*.*1* [48] with four independent chains of 6,000 iterations each and a warm-up period of 3,000 iterations each (producing 12,000 useable samples overall). The categorical predictors were condition (reversal vs. control), design (within vs. between), and initial-choice identification (correct vs. incorrect). The continuous predictor was expected choices—the proportion of higher-beauty choices made for the 4 trials involving an average-beauty painting within each block. Prior to fitting, the categorical predictors were centred (i.e., coded as -1 or 1) and the continuous predictor was scaled (i.e., mean centered and standardized according to its standard deviation). Uninformative priors were employed for both the intercept and slopes—representing a normal distribution with a logit-transformed mean of -1 and standard deviation of 1 (reflecting a weak expectation that reversals would occur less than 50% of the time) and a logit-transformed mean of 0 and standard deviation of 1.5, respectively. Due to the small number of replications per subject, mildly regularizing priors were also placed on the random effects using the decov function from the rstanarm package with a regularization constant of 2 and a scale constant of 3. Sensitivity analyses revealed our conclusions to be robust to a variety of uninformative priors.

Convergence of each model was confirmed visually as well as using the R-hat statistic (in all cases R-hat ≈ 1 and *N*_*Effective*_ > 2000, indicating convergence [49, 50]). Where appropriate, models included both subject-level random intercepts (modelling individual differences in the absolute propensity to reverse) and slopes (modelling individual differences in the magnitude of each slope coefficient). Item-level random effects were not included because only 4 critical painting pairs were used. Experiment-level random intercepts and/or slopes were also excluded because only 3 experiments were conducted. We therefore report the equivalent of a fixed-effect meta-analysis of the individual participant data. Including item-level random effects and/or experiment-level random effects had minimal impact on model parameters and did not affect our conclusions.

Our primary interest was in understanding the impact of our experimental manipulation, thus condition was included in each model. However, we were also interested in understanding the potential influence of study design, initial-choice-identification, and expected choices. It was not possible to include all of these predictors in a single model given the design and data constraints. Instead, each model included condition along with a single other moderator, plus the relevant two-way interaction. We fit each model with the maximal random structure, but convergence issues for the model that included the initial-choice-identification predictor required us to fit the maximal structure possible given the data instead (in this case a random intercept and slope for condition only). Models included all data measuring the relevant variables. Simple effect estimates were calculated at the midpoint of other variable within that model.

**Fig 2:**
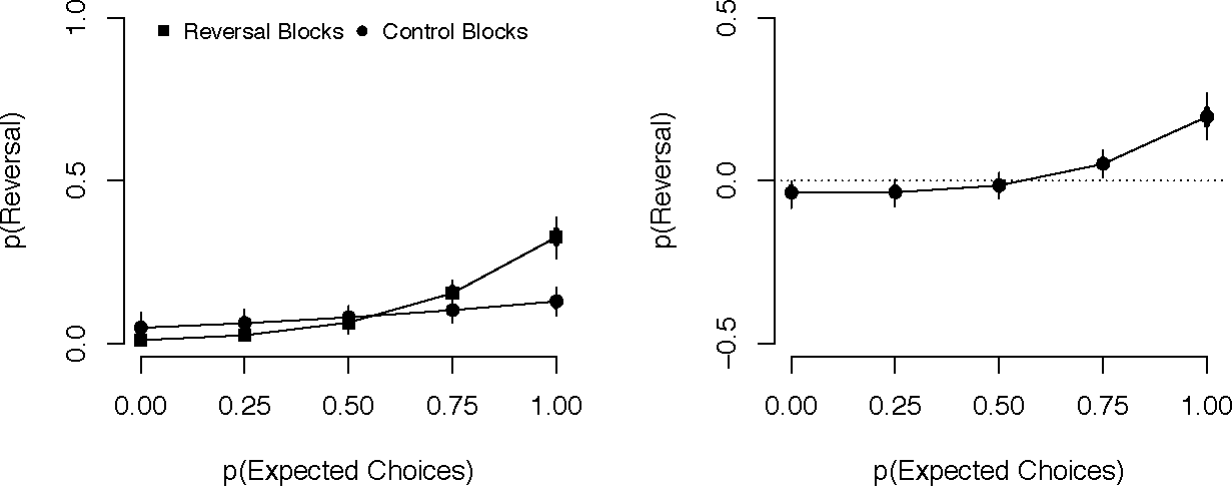
Probability of preference reversals as a function of condition and expected choices. The left panel contains the raw predicted values and the right panel contains difference scores (reversal–control) for each level of expected choices. Error bars represent 95% (thin lines) and 50% (thick lines) HDIs.

The first meta-analysis examined the probability of a reversal as a function of condition and expected choice rate in a Bayesian logistic mixed-effects model. Counterfactual predictions are depicted in Fig 2 and logit-transformed model coefficients are provided in Table 1; each plot represents mean performance (back-transformed into percentages) predicted for an average participant within the model. Fig 2 clearly depicts an overall tendency for participants to reverse their aesthetic choice more frequently in a reversal block, *M* = 14.4%, *HDI*_*95%*_ [9.6%, 18.3%], than in a control block, *M* = 10.0%, *HDI*_*95%*_ [6.3%, 13.2%], difference = 4.4%, *HDI*_*95%*_ [0.0%, 8.2%]. Importantly, Fig 2 also clearly shows that this reversal effect occurs only for participants who consistently made the expected higher-beauty choices during the contrast trials. Indeed, the effect of condition was 14.7%, *HDI*_95%_ [8.8%, 21.0%] greater for participants whose expected choice rate was 75 or 100% than for participants whose expected choice rate was only 0 or 25%.

**Table 1:** Parameter estimates for the Bayesian logistic mixed-effects models pertaining to each moderator.

	Meta of Condition with Expected Choices	Meta of Condition with Design	Meta of Condition with Initial-Choice Identification
Fixed Effects			
Intercept	-1.99 [-2.38, -1.72]	-1.94 [-2.29, -1.67]	-0.97 [-1.30, -0.67]
ß_Condition_	0.21 [0.01, 0.40]	0.32 [0.10, 0.56]	0.16 [-0.11, 0.45]
ß_Moderator_	0.70 [0.48, 0.99]	-0.01 [-0.18, 0.15]	1.77 [1.41, 2.20]
ß_Condition x Moderator_	0.40 [0.18, 0.63]	-0.13 [-0.30, 0.04]	0.00 [-0.30, 0.32]
Random Effects (SD)			
Intercept	0.21 [0.00, 0.76]	0.43 [0.00, 0.90]	0.75 [0.02, 1.32]
ß_Condition_	0.22 [0.00, 0.79]	0.53 [0.00, 1.03]	0.60 [0.00, 1.18]
ß_Moderator_	0.20 [0.00, 0.52]	–	–
ß_Condition x Moderator_	0.20 [0.00, 0.5]	–	–

Notes. Variables were centred prior to analysis (see text) and parameters are provided in logit-space. Values in parentheses represent 95% HDIs.

**Fig 3:**
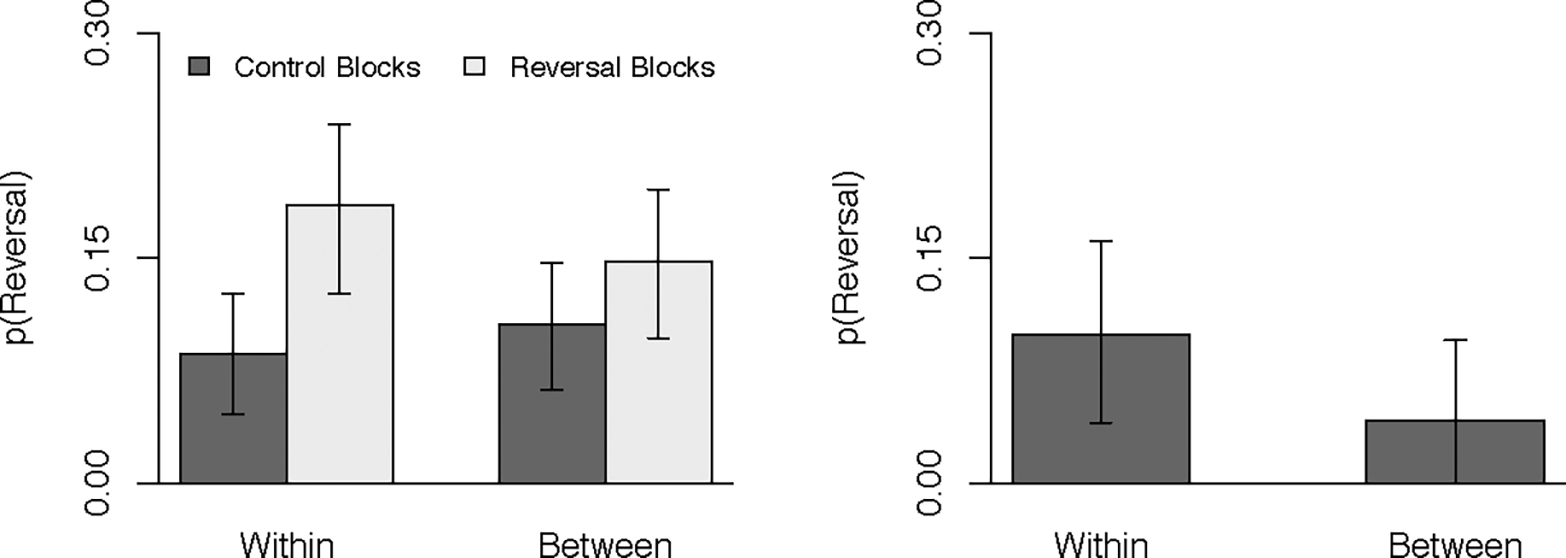
Probability of preference reversals as a function of condition and design. The left panel contains the raw predicted values and the right panel contains difference scores (reversal–control) for each level of design. Error bars represent 95% (thin lines) and 50% (thick lines) HDIs.

The second meta-analysis was fit in the same manner, but now included design in place of expected choices. Counterfactual predictions are depicted in Fig 3 and logit-transformed model coefficients are provided in Table 1. The reversal effect was again apparent, with more preference reversals occurring in reversal blocks, *M* = 16.6%, *HDI*_*95%*_ [11.9%, 20.8%], than in control blocks, *M* = 9.5%, *HDI*_*95%*_ [5.8%, 12.9%], difference = 7.1%, *HDI*_*95%*_ [2.5%, 11.6%]. The condition effect was numerically larger in the within-subject design (94.1% of the credible values for this difference were positive), but the model failed to credibly exclude the possibility that design had no influence, *M* = 5.7%, *HDI*_*95%*_ [-1.8%, 12.9%].

**Fig 4:**
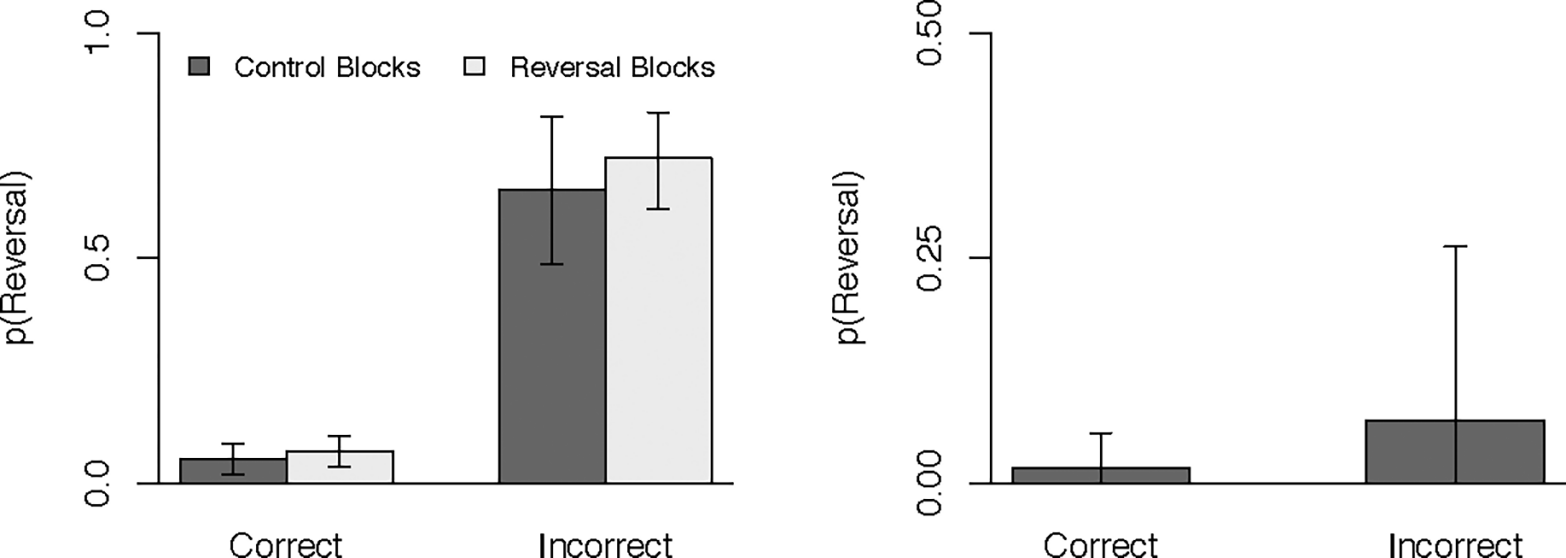
Probability of preference reversals as a function of condition and initial-choice-identification accuracy. The left panel contains the raw predicted values and the right panel contains difference scores (reversal–control) for each level of accuracy. Error bars represent 95% (thin lines) and 50% (thick lines) HDIs.

The third meta-analysis was fit in the same manner, but now included initial-choice identification as the moderator. Experiment 1A was excluded because it did not measure this variable. Due to convergence issues, we were unable to include random effects for the main effect of initial-choice-identification or the interaction–so this model included only the random intercept and slope for condition. Counterfactual predictions are depicted in Fig 4 and logit-transformed model coefficients are provided in Table 1. Despite excluding a sizable portion of our sample, the reversal effect was still evident, although the difference between reversals blocks, *M* = 30.7%, *HDI*_*95%*_ [23.7%, 38.3%], and control blocks, *M* = 24.4%, *HDI*_*95%*_ [15.7%, 33.2%], was no longer credible in the reduced sample, difference = 6.3%, *HDI*_*95%*_ [-4.4%, 16.9%]. Of greater interest in this meta-analysis is the very large main effect of initial-choice identification. Participants were far more likely to reverse if their initial-choice identification was incorrect, *M* = 68.9%, *HDI*_*95%*_ [59.0%, 79.1%], than if it was correct, *M* = 6.0%, *HDI*_*95%*_ [3.2%, 9.0%], difference = 62.7%, *HDI*_*95%*_ [51.6%, 73.9%]. The interaction between condition and initial choice identification was not at all credible, difference = 5.3%, *HDI*_*95%*_ [-26.0%, 13.7%]. However, this might be due to the small number of trials in which participants failed to recall their initial choice (8% for the control blocks and 17% for the experimental blocks). As a potential avenue for future study, the pattern pointed toward a larger effect of condition for trials in which participants made an incorrect initial-choice identification at the end of the experiment.

## Discussion

We introduced a novel paradigm for rapidly inducing reversals in choices, using contrast manipulations previously shown to influence aesthetic judgments [34–35]. Meta-analyses confirmed that choice reversals between pairs of average-beauty abstract paintings were more likely following reversal blocks than following control blocks. Our paradigm thus succeeded in inducing reversals for the same choice in the same task by the same participant—separated by only 6 intervening choices. Notably, our paradigm shows that under the right conditions, it is possible to induce choice reversals indirectly through context manipulation rather than by manipulating the target choices directly, such as by varying their relative durations [25–26]. Our paradigm thus complements an existing set of paradigms for inducing and studying choice reversals [13–26]. We implemented the contrast paradigm in both within-subject (Experiment 1) and between-subject (Experiment 2) designs. We did not find a credible effect of design in our meta-analysis, but the effect was certainly not larger in the between-subject design. Given that the within-subject design controls for individual differences (because each participant serves as their own control), it may be preferable in most situations.

Two other aspects of our results were novel. First, the reversal effect was related to the number of expected choices participants made within the blocks. Second, and replicating recent findings using a different paradigm [43], reversals were more likely for participants who incorrectly identified their initial choices later in the experiment. Thus, memory for initial choices was related to choice reversals. We next consider each of these key aspects of our study in more detail.

### The role of expected choices in modulating choice reversals

The meta-analysis showed that reversal effect was present only when participants’ choices were consistently biased in the expected manner within the reversal block (see Fig 1). Merely being exposed to the reversal block pairs was not sufficient to produce a reversal effect—participants’ choices had to be successfully biased for the effect to occur. Indeed, the reversal effect was essentially limited to participants who always chose the expected painting. Because this function was not predicted, we did not set a “proportion of expected choices cut-off” for analysis of the reversal effect in our meta-analyses. However, as Fig 1 shows, the reversal effect was substantial when participants’ choices were effectively biased by the contrast trials.

### The role of memory in modulating choice reversals

The meta-analysis also showed that participants who misremembered their initial choice were much more likely to later reverse their choice—regardless of condition. This finding dovetails nicely with other recent evidence suggesting an important role for episodic memory for past choices in creating preferences [41, 43]. A critical direction for future research will be to unpack the relationship between memory and choice reversals. People who cannot recall their initial choices cannot experience cognitive dissonance for those choices. Perhaps this frees them from feeling pressure to be consistent, thus increasing the likelihood of choice reversals. Conversely, participants who misremember their initial choice may feel pressure to be consistent, ironically leading them to reverse their choice.

One potential concern with our initial-choice identification measure is that it may not have accurately captured participants’ memories, given it was collected at the end of the main experiment. It may have been difficult for participants to remember their initial choice given our method. Our choice memory measure was similar to the measure used in previous work [41, 43], except our measure involved presenting the stimuli in their original pairs rather than individually. In their fMRI study, Chammat et al. [41] found that the behavioral relationship between memory assessed after the main experiment and choice-induced preference change was also present neurally; greater BOLD activation in the hippocampus was found in the rating-choice-rating condition compared to the rating-rating-choice condition, but only for remembered items. They obtained further evidence of a link between hippocampal activity and choice memory using intracranial electrophysiological recordings of hippocampal event-related potentials in epileptic patients while they completed the task. The similarity in memory measures across their studies and ours supports our claim that our initial-choice identification measure reflected participants’ memory for items and choices, even though it was collected at the end of the experiment. However, future research should examine whether the effect obtains when memory for choice 1 is measured immediately after choice 1 and/or 2.

### Exploring the contrast paradigm

There are many potential ways to extend the contrast paradigm, including exploring how choice reversals are affected by adding a delay between choices and by adding more repetitions of the reversal block trials [51]. The paradigm might also yield larger reversal effects if a participant’s own ratings were used to select the paintings for the blocks, as was the case in Salti et al. [43], rather than using piloting norms to select items. Other methods of amplifying the reversal effect could also be explored. For example, a cognitive load task may reduce participants’ ability to exert cognitive control and/or to experience cognitive dissonance [1, 52], in turn rendering them more susceptible to contrast effects.

Whether the contrast paradigm induces reversals for other types of stimuli also warrants consideration. In the aesthetics domain, in light of evidence that abstract and representational artworks are evaluated differently [12], it would be worth testing whether a reversal effect occurs for representational paintings. Stimuli of interest to marketers, such as logos, brands, and products could also be used. For example, Zellner, Allen, Henley, and Parker [53] induced a contrast effect on people’s preferences for juice samples. Our paradigm could easily be adapted to examine choice reversals for actual samples of products.

### Implications for aesthetics research

Sets of similar paintings, or of other types artworks, are often curated and displayed together in exhibits, galleries, books, or albums. Our research adds to a growing body of evidence that examines how judgments about artworks are influenced by the other artworks in a given context [32, 35, 54]. Such findings will prove helpful for constraining accounts of aesthetic judgments [55–58]. For example, the finding of contextual influences on aesthetic choices indicates that the objective qualities of stimuli cannot fully explain the basis of aesthetic preferences [12]. A stimulus that is deemed more beautiful than another stimulus in one context may be deemed less beautiful in another context.

One limitation of our study for researchers interested in aesthetics is that we did not determine which properties of the paintings we selected led them to be consistently rated as low, average, or high beauty in our pilot studies. A related study from our lab examined which subjective ratings and objective stimulus properties predict beauty ratings based on the paintings in our initial stimulus set [59]. Greater liking of abstract paintings was predicted by higher subjective ratings of emotionality, and by higher objective entropy scores (i.e., a computed index of the unpredictability or disorder of the pixels in a painting image).

### Conclusion

The present study contributes to our understanding of the factors that modulate subjective choice reversals. The contrast paradigm highlights the curious instability of choices among similar alternatives, as well as the influence of local context on choices. We also reported new evidence that memory for initial choices can offer a protective factor against subsequent choice reversals. These preliminary findings lay the groundwork for extending the contrast paradigm in ways that will inform future research on preferences, choices, and decision making—research that should prove informative for marketers, economists, and psychologists alike.

## Supporting information

S1 Table Counterbalancing conditions in Experiments 1 and 2. (DOCX)
