# Supplementary material for: Inducing preference reversals in aesthetic choices for paintings: Introducing the contrast paradigm
Source: PLoS One. 2018 Apr 19;13(4):e0196246. doi: 10.1371/journal.pone.0196246 (PMC5908093; doi:10.1371/journal.pone.0196246)
Supplement: S1 Table — (DOCX) [file pone.0196246.s001.docx]

| **Exp 1 Counterbalance** | **Exp 2 Counterbalance** | **Reversal 1** | **Reversal 2** | **Control 1** | **Control 2** |
| --- | --- | --- | --- | --- | --- |
| 1= Reversal1 Control2  2= Control2 Reversal1 | 1= Reversal1 Reversal2  2= Reversal2 Reversal1  3= Control1 Control2  4= Control2 Control1 | **A1 A2**  H1 L1  H2 L2 | **A3 A4**  H3 L3  H4 L4 | **A1 A2**  *A7H1 A8L1*  *A7H2 A8L2* | **A3 A4**  *A5H3 A6L3*  *A5H4 A6L4* |
| 3= Reversal1 Control2  4= Control2 Reversal1 |  | **A3 A4**  H1 L1  H2 L2 | **A1 A2**  H3 L3  H4 L4 | **A3 A4**  *A5H1 A6L1*  *A5H2 A6L2* | **A1 A2**  *A7H3 A8L3*  *A7H4 A8L4* |
| 5= Reversal1 Control2  6= Control2 Reversal1 | 5= Reversal1 Reversal2  6= Reversal2 Reversal1  7= Control1 Control2  8= Control2 Control1 | **A5 A6**  H1 L1  H2 L2 | **A7 A8**  H3 L3  H4 L4 | **A5 A6**  *A3H1 A4L1*  *A3H2 A4L2* | **A7 A8**  *A1H3 A2L3*  *A1H4 A2L4* |
| 7= Reversal1 Control2  8= Control2 Reversal1 |  | **A7 A8**  H1 L1  H2 L2 | **A5 A6**  H3 L3  H4 L4 | **A7 A8**  *A1H1 A2L1*  *A1H2 A2L2* | **A5 A6**  *A3H3 A4L3*  *A3H4 A4L4* |

**S1 Table. Counterbalancing conditions in Experiments 1 and 2.**

Note: A = average-beauty painting; H = high-beauty painting; L = low-beauty painting. Each of 8 counterbalances are listed as pairs of blocks for Experiments 1 and 2. Reversal and control block painting assignments show target average pairs in bold, and the high and low paintings assigned to the contrast trials. In control blocks, high and low paintings were paired with average paintings other than the target pairs. In Experiment 1A, 8 participants completed each counterbalance. In Experiment 1B, 28 participants completed each counterbalance. In Experiment 2, 48 participants completed each counterbalance.
